# Social Capital, Life Meaning, and Depressive Symptoms Among Older Adults in China: The Moderating Roles of Digital Social Participation and Digital Social Interaction

**DOI:** 10.3390/bs16101833

**Published:** 2026-10-07

**Authors:** Jingfang Liu, Chengyang Fu, Dingyun Lou

**Affiliations:** School of Management, Shanghai University, Shanghai 200444, China

**Keywords:** older adults, depressive symptoms, social capital, digital social participation, digital social interaction, intergenerational interaction, interpersonal trust, moderated mediation

## Abstract

Population aging and digitization are two defining trends in China, yet how digital forms of social participation and interaction condition the association between social capital and older adults’ mental health remains unclear. Using cross-sectional data from the 2022 China Family Panel Studies (*N* = 4394 adults aged 60 years and above), this study examined whether two bounded indicators of social capital—intergenerational interaction frequency and interpersonal trust—are associated with depressive symptoms, whether life meaning carries part of those associations, and whether two forms of digital social engagement moderate them. A moderated mediation model with five regression equations was estimated by survey-weighted least squares, with design-based standard errors and confidence intervals obtained by bootstrap replication of primary sampling units. Both intergenerational interaction frequency (β = −0.238, *p* < 0.001) and interpersonal trust (β = −0.756, *p* < 0.001) were negatively associated with depressive symptoms, and the indirect associations through life meaning were −0.047 (95% CI −0.072 to −0.021) and −0.163 (95% CI −0.252 to −0.074) respectively, accounting for 19.6% and 21.6% of the corresponding total associations. Both digital measures moderated associations within the model and in opposite directions: the negative association between interpersonal trust and depressive symptoms was stronger among respondents who used WeChat (interaction β = −0.719, *p* = 0.011), whereas the positive association between intergenerational interaction frequency and life meaning was weaker among those who shared more often on WeChat Moments (interaction β = −0.035, *p* < 0.001). These findings suggest that digital social participation and digital social interaction are not merely independent correlates of older adults’ mental health but conditions under which the associations between specific social capital indicators and mental health take different forms.

## 1. Introduction

Population aging is a defining demographic trend in China, characterized by its unprecedented scale, rapid pace, and deepening structural transformation. The mental health of older adults has become a central concern for public health, social welfare, and sustainable development. Within the framework of Healthy China and active aging policies, the scope of geriatric health assessment has expanded beyond physical health to encompass psychological well-being and social adaptation. Mental health constitutes the core of quality of life in later years and serves as a critical lever for advancing healthy aging. However, with evolving family structures and the increasing prevalence of solitary and empty-nest living arrangements, the psychological burden on older adults in China continues to grow. Depressive symptoms, loneliness, and diminished self-worth have become pressing challenges, calling for a deeper understanding of the mechanisms that shape mental health in older populations.

The rapid advancement of digital technologies has ushered society into an era of pervasive digitization. Mobile internet, smart devices, digital social networking, online learning, and digital entertainment have reshaped daily life, modes of social interaction, and channels for accessing resources. While digitalization offers new opportunities for enriching older adults’ lives—broadening their social engagement and facilitating access to public services—older adults remain disproportionately affected by the digital divide due to physiological decline, cognitive limitations, insufficient digital literacy, and constrained living environments. The contemporary digital divide among older adults is no longer a simple matter of physical access to devices and networks. Rather, it has evolved into a multidimensional phenomenon encompassing differential capabilities in digital tool use, depth of engagement across digital contexts, and quality of digital social interaction (van Dijk, 2006). Older adults who are marginalized in the digital realm face deepening disadvantages in social participation, interpersonal interaction, and resource acquisition—inequalities that can profoundly affect their emotional well-being and psychological state.

Among the protective factors for mental health in later life, social capital occupies a central position. Social capital theory distinguishes two dimensions—structural and cognitive (Coleman, 1988; Uphoff, 2000); the detailed definitions and the two indicators used in this study are set out in Section 1.1.1. The framework is organized around two primary theories. Social capital theory defines the structural and cognitive dimensions that structure the independent variables, and logotherapy establishes the theoretical status of life meaning as the mediator. Four further theories are drawn on in a supporting capacity: the stress-buffering model, which motivates the direct associations between social capital and depressive symptoms; social identity theory, which motivates the link between social capital and life meaning; and channel complementarity and media substitution theory, which inform the expected direction of the two moderating effects. A large body of evidence shows that rich social capital provides emotional solace and instrumental support, buffering the negative psychological impact of aging-related losses. Zhou et al. (2022) confirmed that social capital exerts a significant protective effect against depressive symptoms among older adults. Z. Xu et al. (2022) further demonstrated that social capital is positively associated with subjective well-being in older adults across family, workplace, community, and societal levels, with family support and community participation being particularly effective in alleviating loneliness and depression.

In the structural dimension, intergenerational interaction—the frequency and quality of contact between older adults and their children—has been identified as a fundamental form of social capital. Studies using longitudinal data from the China Health and Retirement Longitudinal Study (CHARLS) have shown that living arrangements that facilitate parent–child contact exert a causal, positive influence on older adults’ mental health (Q. Xu, 2018). J. Tian and Li (2023) found that both family and friend networks contribute to mental health in older adults, with internet use playing a moderating role that strengthens family ties. In the cognitive dimension, interpersonal trust—the generalized expectation that others can be relied upon—has been shown to be a critical component. Kawachi and Berkman (2001) systematically reviewed the protective mechanisms of social networks and social trust for older adults’ mental health, demonstrating that neighborhood trust and community norms of reciprocity reduce the risk of depression and anxiety by enhancing individuals’ sense of security and belonging. In a prospective study, Kim et al. (2012) reported that higher levels of interpersonal trust and perceived reciprocity were associated with a roughly 40% lower risk of incident depression over a two-year period.

Regarding the pathway through which social capital may contribute to mental health, life meaning—a sense of purpose and significance in one’s existence—has received growing attention. Vissers et al. (2024) systematically reviewed the role of meaning in late life, highlighting social connection and interaction as central factors in maintaining and enhancing life meaning. From a logotherapy perspective, a qualitative study of older adults living in long-term care found that spirituality and life meaning served as resources for coping with the difficulties of that setting (Miranda et al., 2020). These findings suggest that life meaning may function as a key psychological pathway through which social capital protects against depression.

Despite this rich body of work, two important gaps remain in the literature. First, most existing research on social capital and older adults’ mental health has focused on the direct effects of social capital or on traditional mediation pathways, without adequately accounting for how the digital era has reconfigured the boundary conditions under which social capital works. The digital divide is rarely incorporated as an important contextual variable into analytical frameworks linking social capital to mental health. Second, digital divide research has largely remained confined to the technological domain, focusing on access disparities or aggregate usage metrics, and has not established a systematic dialogue with the social capital and mental health literature. In reality, digital social participation and digital social interaction may operate not only as independent correlates of mental health but as conditions that moderate the associations of social capital with mental health. The extent to which older adults engage digitally may therefore condition how strongly social capital is associated with their mental health.

### 1.1. Hypothesis Development

#### 1.1.1. Social Capital and Depressive Symptoms

Social capital theory conceptualizes social capital as the resources embedded in social networks and governed by norms of reciprocity (Coleman, 1988; Uphoff, 2000), conventionally distinguishing between structural and cognitive dimensions. Structural social capital refers to objective network ties, interaction behaviors, and role relationships, while cognitive social capital refers to subjective perceptions of trust, norms, and shared values. In the context of aging, older adults’ social networks shrink, with family and close social ties becoming the primary channels for accessing social resources (Carstensen, 1992; Lang & Carstensen, 1994). Accordingly, this study operationalizes structural social capital as intergenerational interaction frequency—the frequency of face-to-face contact between older adults and their children—and cognitive social capital as interpersonal trust—whether the respondent believes that most people can be trusted. We treat these as bounded, single-indicator measures of the structural and cognitive dimensions rather than as comprehensive measures of social capital, and we restrict our conclusions accordingly.

According to the stress-buffering model (Bovier et al., 2004; Cohen & Wills, 1985), when individuals confront stressful events, external social support and internal psychological resources function as buffers that attenuate the negative impact of stressors on physical and mental health. In later life, older adults face objective stressors such as physical decline and social role loss. As structural social capital, higher intergenerational interaction frequency means that older adults receive more substantial caregiving support and emotional comfort from their children; this objective network interaction directly buffers life stressors, reducing feelings of helplessness and depression risk. As cognitive social capital, older adults with higher interpersonal trust tend to hold more positive cognitive schemas, approaching life changes with openness and optimism, and are more willing to seek external help when encountering difficulties. This trust-based sense of security and belonging effectively resists the accumulation of negative affect, thereby alleviating depressive symptoms. We therefore hypothesize the following:

**H1a.**  *Interpersonal trust is negatively associated with depressive symptoms in older adults.*

**H1b.**  *Intergenerational interaction frequency is negatively associated with depressive symptoms in older adults.*

#### 1.1.2. The Mediating Role of Life Meaning

Social identity theory posits that individuals categorize themselves into specific groups, internalize group membership, and derive emotional support and value significance from group belonging, thereby forming social identity and self-concept (Spears, 2021; Tajfel & Turner, 2000). For older adults, the accumulation of social capital is intimately tied to the construction of social identity (Haslam et al., 2009). Specifically, intergenerational interaction frequency, as an indicator of structural social capital, reflects the degree of older adults’ actual participation in family and intergenerational networks. Frequent intergenerational interaction enables older adults to continuously perceive emotional support and role affirmation from their children, reinforcing their membership in the family in-group, which in turn generates a deep sense of social identity and life meaning. Similarly, interpersonal trust, as the core dimension of cognitive social capital, reflects older adults’ cognitive appraisal of the trustworthiness of others. Higher interpersonal trust means that older adults are more inclined to categorize those around them as trustworthy in-group members, facilitating social categorization and group belonging in interpersonal exchanges, thereby forming positive social identity. This positive cognitive connection enables older adults to recognize themselves as belonging to a mutually trusting, reciprocal social network, with the emotional support and value affirmation conferred by group membership providing transcendent existential meaning, thereby enhancing life meaning.

Furthermore, logotherapy emphasizes that the pursuit of life meaning is the most fundamental human motivation; individuals who perceive clear meaning in life possess the psychological resilience to confront frustration, emptiness, and negative affect (Botîlcă, 2024; Russo-Netzer & Ameli, 2021). In later life, older adults often face role loss, physical decline, and social network contraction, rendering them vulnerable to feelings of emptiness that can induce or exacerbate depressive tendencies. Life meaning, as a deep positive psychological resource, constitutes a critical line of defense against depression. When older adults possess a high level of life meaning, they are able to view the aging process from a more constructive and accepting perspective, drawing intrinsic motivation from daily family interactions and social participation to sustain self-worth and psychological equilibrium. This clear sense of meaning effectively buffers the feelings of loss and meaninglessness associated with aging, blocking the accumulation of negative affect and thereby significantly alleviating depressive symptoms. We therefore hypothesize the following:

**H2a.**  *Life meaning mediates the relationship between interpersonal trust and depressive symptoms.*

**H2b.**  *Life meaning mediates the relationship between intergenerational interaction frequency and depressive symptoms.*

**H3.**  *Life meaning is negatively associated with depressive symptoms in older adults.*

#### 1.1.3. The Moderating Role of Digital Social Engagement

Digital inequality is conventionally described as unfolding in successive stages, from the access divide through the usage divide to the outcome divide (van Dijk, 2006). We retain this framework as background, but our two indicators are deliberately narrower than it. Digital social participation (whether the respondent used WeChat in the past year) and digital social interaction (how often the respondent shared content on WeChat Moments) are measures of digital social engagement. The first captures whether an older adult engages in online social networking at all; the second captures how actively they share within that network. They are not measures of the access, skills, usage or outcome divides, and we do not interpret them as such. Our analytical interest lies in whether these two forms of digital social behavior condition the associations between social capital and mental health, not in locating respondents within a staged account of digital inequality.

We propose that the two moderating effects differ because the two indicators bear on different processes rather than because they belong to different dimensions of digital inequality. Both are behavioral measures of what older adults do online—whether they use WeChat at all and how often they share within it—and we draw no inference from them about psychological acceptance or cognitive accessibility. The first pairing concerns the arena in which a disposition toward others is expressed: digital social participation brings interpersonal trust into an online social setting, where it may be reinforced or left unreciprocated. The second concerns the allocation of activity: sharing online and interacting with one’s children are both ways of spending time and emotional investment, and the maintenance of meaning through social activity may depend on how the two are combined. Interpersonal trust and life meaning are not interchangeable in this respect, since the first concerns how a cognitive disposition is expressed in social relations, whereas the second is sustained through ongoing activity.

A further consideration led us to the same pairing. Depressive symptoms and life meaning are not mirror images: the alleviation of negative affect is commonly understood to depend on cognitive appraisal (Folkman, 2013), whereas life meaning is sustained through ongoing social activity (Havighurst, 1953). If the two indicators of digital social engagement bear on different psychological processes in this way, participation should act more directly on the interpersonal trust–depression association, and interaction should act more directly on the intergenerational contact—meaning association. This reasoning is what led us to specify H4a and H4b as set out below; we return to its status in Section 4.

With the moderating pathways established, the direction of each effect can be further explained by the theory of channel complementarity and media substitution theory. According to channel complementarity theory, different media channels are not in a zero-sum relationship in terms of function and utility; rather, they can complement and reinforce each other (Dutta-Bergman, 2004). Older adults with high interpersonal trust tend to have strong motivation for social engagement; digital social participation expands a new arena for trust expression and identity construction for them. The complementarity of online trust networks and offline cognitive resources may reinforce older adults’ perceived social support and strengthen the negative association between interpersonal trust and depressive symptoms. Conversely, according to media substitution theory, individuals’ time and energy are finite; deep engagement with one medium necessarily crowds out investment in another (Dimmick, 2002; Kaye & Johnson, 2003). Digital social interaction, as deep online behavior, involves high-frequency online interaction that may substitute for or diminish the time and emotional investment devoted to offline intergenerational companionship, rendering in-person intergenerational interaction perfunctory or degraded in quality. If so, digital social interaction would weaken the association between intergenerational interaction frequency and life meaning. We therefore hypothesize the following:

**H4a.**  *Digital social participation moderates the relationship between interpersonal trust and depressive symptoms such that the negative association is stronger among older adults who participate in digital social networks.*

**H4b.**  *Digital social interaction moderates the relationship between intergenerational interaction frequency and life meaning such that the positive association is weaker among older adults with more frequent digital social interaction.*

**Figure 1:**
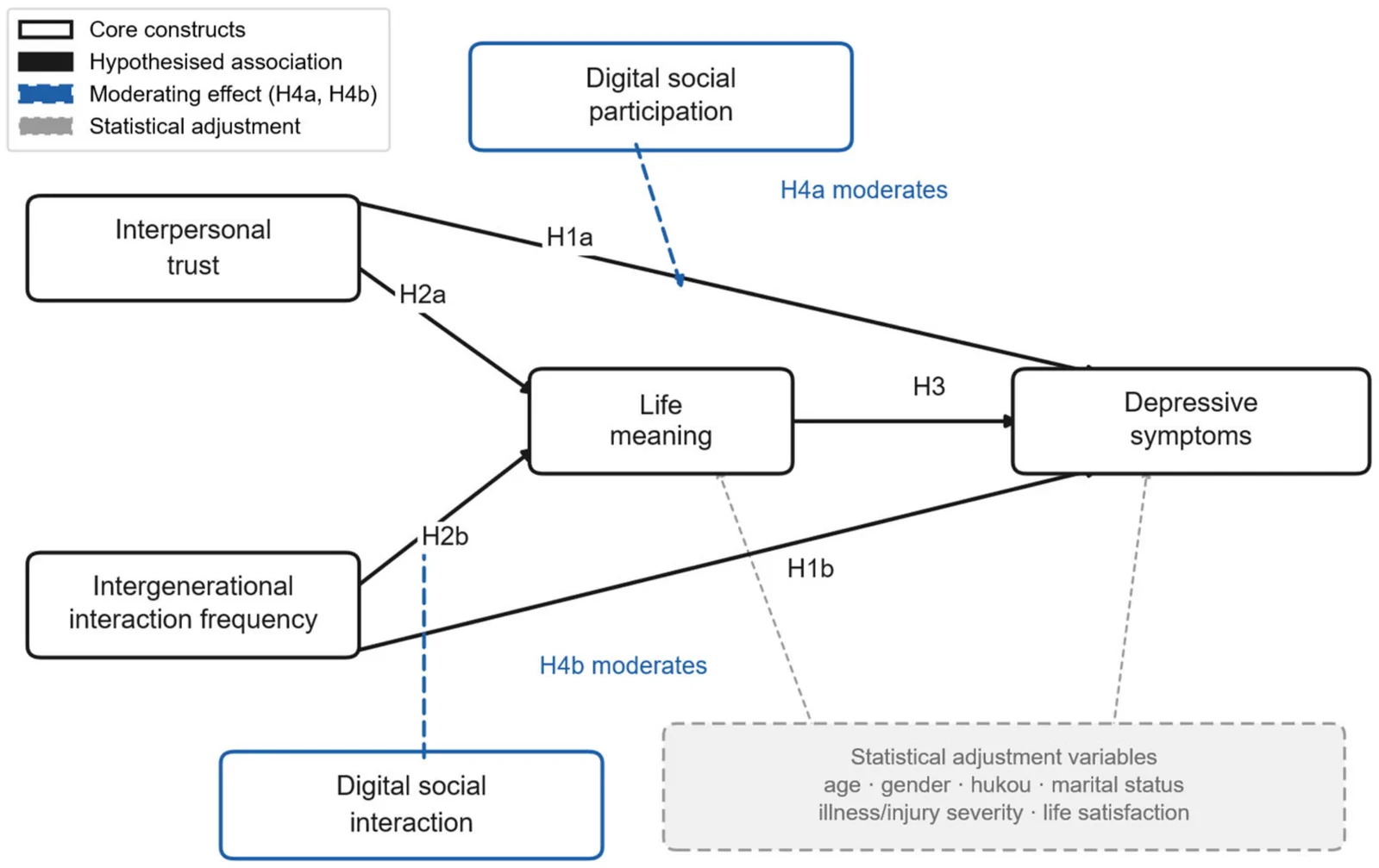
Conceptual model of the association between social capital and depressive symptoms in older adults, with digital social participation and digital social interaction as moderators. Solid arrows denote hypothesised associations; dashed blue arrows denote the moderating effects proposed in H4a and H4b; the shaded block denotes statistical adjustment. Digital social participation is measured by whether the respondent used WeChat in the past year and digital social interaction by how often the respondent shared content on WeChat Moments. Age, gender, hukou, marital status, illness/injury severity and life satisfaction are entered as covariates in every estimated equation, including those for life meaning and for depressive symptoms.

The full conceptual model is presented in Figure 1.

### 1.2. The Present Study

To address the identified gaps, this study constructs an integrated analytical framework that examines how structural and cognitive social capital relate to depressive symptoms in older adults, and whether two forms of digital social engagement condition these associations. Drawing on nationally representative data from the 2022 wave of the China Family Panel Studies (CFPS) (Xie & Hu, 2014), we focus on older adults aged 60 years and above (*N* = 4394 with valid sampling weights) and specify a moderated mediation model with five interconnected regression equations. Several psychological mediators of the social-relationship–depression association have already been established: loneliness (Hou et al., 2021; G. Tian et al., 2025), perceived social support (Gautam et al., 2024; Y. Liu et al., 2024), self-esteem (Huang et al., 2022; Thoits, 2011), and life satisfaction (dos Santos et al., 2023; Lopez-Zafra et al., 2019). Meaning in life has recently joined them, with longitudinal evidence that perceived social support lowers depression partly through a gratitude–meaning pathway (H. M. Tian et al., 2025) and supporting findings from network and chain-mediation analyses (Yuan et al., 2024; Zhang et al., 2025). Two features distinguish the present study. First, this evidence comes predominantly from adolescent and student samples, leaving the question comparatively open for older adults, whose social networks and sources of meaning are organized differently. Second, these studies establish whether a variable mediates but not whether the mediated pathway is conditional. We ask whether the strength of the indirect association depends on older adults’ digital social participation and digital social interaction.

The framework is built around three layers. First, we examine the direct associations of intergenerational interaction frequency (structural social capital) and interpersonal trust (cognitive social capital) with depressive symptoms, as measured by the CES-D-8 scale. Drawing on the stress-buffering model (Bovier et al., 2004; Cohen & Wills, 1985), we hypothesize that both dimensions of social capital serve as buffers that attenuate the negative impact of aging-related stressors on psychological well-being. Second, we investigate whether life meaning mediates these relationships. Grounded in social identity theory (Spears, 2021; Tajfel & Turner, 2000) and logotherapy (Botîlcă, 2024; Russo-Netzer & Ameli, 2021), we propose that social capital is associated with a stronger sense of purpose and belonging, which is in turn associated with fewer depressive symptoms. Third, we introduce two forms of digital social engagement as boundary conditions. Drawing on van Dijk (2006) on digital inequality, we distinguish between digital social participation—whether an older adult uses WeChat at all—and digital social interaction—how frequently they share content on WeChat Moments—and examine how each moderates distinct pathways in the model. Specifically, guided by the theory of channel complementarity (Dutta-Bergman, 2004) and media substitution theory (Dimmick, 2002; Kaye & Johnson, 2003), we hypothesize that digital social participation strengthens the negative association between interpersonal trust and depressive symptoms, whereas digital social interaction weakens the positive association between intergenerational interaction frequency and life meaning.

This study makes three primary contributions. First, it constructs an integrated framework linking structural and cognitive social capital to depressive symptoms in older adults, with life meaning as a mediator and two forms of digital social engagement as moderating conditions, thereby extending the theoretical understanding of how social capital works in the digital era. Second, it identifies asymmetric moderating effects of the two forms of digital social engagement: digital social participation strengthens the negative association between interpersonal trust and depressive symptoms, whereas digital social interaction weakens the positive association between intergenerational interaction frequency and life meaning. This asymmetry suggests that digital social engagement is not a monolithic influence but a differentiated contextual one whose associations depend on which form of social capital each indicator concerns. Third, by positioning digital social engagement not merely as an independent antecedent of mental health but as a contextual condition that moderates how social capital is associated with mental health, this study bridges the social capital, digital divide, and mental health literatures, which have largely developed in isolation.

## 2. Materials and Methods

### 2.1. Data Source

The data used in this study were drawn from the CFPS, administered by the Institute of Social Science Survey at Peking University (Xie & Hu, 2014). The CFPS is a nationally representative, large-scale, multidisciplinary longitudinal survey that collects data at the individual, household, and community levels to document changes in China’s economy, society, demographics, education, and health. The survey covers 25 provinces across China and employs a multistage, implicitly stratified, probability-proportional-to-size sampling design, yielding samples with strong national representativeness and longitudinal comparability.

This study focuses on the complex relationships among the digital divide, social capital, and mental health in older adults. The CFPS was selected as the data source primarily because of its strong alignment with the measurement requirements of our core variables. First, in the mental health domain, the questionnaire measures respondents’ depressive symptoms using the Center for Epidemiologic Studies Depression Scale (CES-D), providing a reliable basis for measuring the dependent variable. Second, in the social capital domain, CFPS records detailed information on respondents’ intergenerational interactions within the family as well as their interpersonal engagement and trust, enabling precise measurement of multidimensional social capital. Third, the questionnaire records respondents’ internet access conditions and digital technology use behaviors, providing direct data support for examining whether digital social participation and digital social interaction moderate the associations of interest. Accordingly, this study uses the latest wave of CFPS data (2022), with a total sample size of 27,001.

### 2.2. Data Processing and Sample Selection

Based on the CFPS 2022 raw data, rigorous screening and cleaning procedures were applied according to the research design and empirical modeling requirements. The specific steps were as follows.

First, the target population was defined, and eligible samples were identified. Consistent with the conventional international and domestic definition of older adults, respondents aged 60 years and above were selected as the study population. After excluding respondents younger than 60 from the raw data, an initial pool of 6860 eligible older adult samples was obtained.

Second, samples with missing values on key variables were excluded. To ensure the stability of the moderated mediation model estimation and the reliability of the findings, each sample was required to have complete observations on all dependent, independent, mediating, moderating, and control variables. The initial pool of 6860 older adult samples was further screened, and invalid samples with missing or unidentifiable information on any variable were removed. After missing value processing, a final valid sample of 4570 respondents was obtained, of whom 4394 (96.15%) had a valid sampling weight and constitute the survey-weighted analysis sample used in all main analyses; the 4570 respondents are retained in unweighted sensitivity analyses.

The pattern of missingness was examined before complete-case selection was applied. Missingness is confined almost entirely to the self-administered questionnaire rather than spread evenly across the data: age and gender are complete, household registration is missing for 0.64% of respondents, and each remaining analysis variable is missing for between 28.8% and 32.4% of the eligible pool (illness severity 28.79%, life meaning 31.44%, life satisfaction 31.34%, interpersonal trust 31.63%, WeChat use 30.73%, sharing frequency 28.82%, depressive symptoms 32.39%). Missingness is also clustered within respondents: 1974 of the 6860 eligible respondents (28.78%) are missing eight or more analysis variables, which corresponds to respondents who did not complete the self-administered questionnaire rather than to item-level non-response. Complete-case analysis therefore rests on the assumption that the excluded values are missing at random given the observed characteristics; because the excluded respondents differ systematically from those retained, that assumption is not innocuous, and the estimates reported below should be read as conditional on it.

Retained and excluded respondents were compared on every analysis variable. Those excluded were substantially older (mean 75.02 vs. 68.34 years, *p* < 0.001), more often female (male 41.97% vs. 51.88%, *p* < 0.001) and less often urban (44.61% vs. 49.78%, *p* < 0.001). Where the focal variables are observable in the excluded group, the differences are likewise pronounced: mean intergenerational interaction frequency was 1.64 versus 2.96 (*p* < 0.001), WeChat use 28.57% versus 46.81% (*p* < 0.001) and sharing frequency 0.22 versus 0.63 (*p* < 0.001). The excluded respondents are therefore less digitally engaged and have less intergenerational contact than those retained. Differences in depressive symptoms (14.09 vs. 13.88) and life meaning (7.36 vs. 7.56) were not statistically significant, but these comparisons rest on only 68 and 133 excluded respondents respectively because the same missingness that removes them from the analysis also removes them from the comparison; they are weakly powered and should not be read as evidence that no selection effect exists. As a sensitivity analysis, the three core equations were re-estimated by multiple imputation by chained equations (20 imputations) on the full eligible sample of 6860 respondents, recovering the 2290 excluded cases. The focal coefficients were essentially unchanged: the association of interpersonal trust with depressive symptoms was −0.671 under multiple imputation versus −0.666 under complete-case analysis, that of intergenerational interaction frequency with depressive symptoms was −0.164 versus −0.165, the association of interpersonal trust with life meaning was 0.208 versus 0.204, that of intergenerational interaction frequency with life meaning was 0.092 versus 0.087, and that of life meaning with depressive symptoms was −0.507 versus −0.504. No focal coefficient moved by more than 0.006, and all retained their direction and significance at *p* < 0.01. The imputation should nevertheless be read as corroborative rather than definitive: the average relative variance increase ranged from 0.62 to 0.64 and the largest fraction of missing information was 0.61, so a substantial share of the information about these coefficients comes from the imputation model rather than from observed data. Two scope limits apply. The imputation covers the three core equations rather than the full moderated mediation model. In addition, because never-married (*n* = 32) and cohabiting (*n* = 22) respondents are too few to support a multinomial imputation model, marital status was imputed in three categories (married, widowed, other) while the four-category specification is retained in every reported model.

Because the CFPS employs a multistage, implicitly stratified probability-proportional-to-size sampling design, the analyses reported below account for the sampling design. We used the 2022 individual cross-sectional weight, the baseline household primary sampling units, and the province of residence as a stratification proxy, since the CFPS does not release a separate stratification variable. Of the 4570 respondents in the analysis sample, 4394 (96.15%) had a valid sampling weight and constitute the weighted analysis sample; the remaining 176 were retained in an unweighted sensitivity analysis. The design comprises 30 strata and 190 design degrees of freedom. Strata containing a single primary sampling unit were retained and centered at the overall mean.

### 2.3. Variables and Measurement

#### 2.3.1. Dependent Variable

The dependent variable is depressive symptoms in older adults, measured using the 8-item Center for Epidemiologic Studies Depression Scale (CES-D-8). The scale comprises eight items asking respondents about the frequency of various feelings or behaviors experienced in the past week: “I felt depressed,” “I felt that everything I did was an effort,” “My sleep was restless,” “I felt happy,” “I felt lonely,” “I enjoyed life,” “I felt sad,” and “I could not get going.” Each item is rated on a four-point scale ranging from rarely or none of the time (<1 day) to most or all of the time (5–7 days). Negatively worded items are scored from 1 (rarely) to 4 (most of the time), while positively worded items are reverse-coded from 4 (rarely) to 1 (most of the time). The eight items are summed to produce a total CES-D-8 score ranging from 8 to 32, with higher scores indicating more severe depressive symptoms.

#### 2.3.2. Independent Variables

The independent variables represent two bounded indicators of social capital, distinguishing the structural dimension, measured by intergenerational interaction frequency, from the cognitive dimension, measured by interpersonal trust. Neither indicator is a comprehensive measure of its dimension; throughout, we refer to them by their specific names to keep the scope of our claims explicit.

Intergenerational interaction frequency is measured by the item “Over the past six months, how often did you see your child(ren)?” Responses range from 0 (never) to 6 (almost every day), corresponding to the categories “never,” “once every few months,” “once a month,” “2–3 times a month,” “1–2 times a week,” “3–4 times a week,” and “almost every day,” respectively. Since respondents may have multiple children, the average frequency of meeting across all children was calculated, with higher values indicating more frequent intergenerational interaction. Co-resident and non-co-resident children are not distinguished: the instrument asks how often the respondent sees each child listed, applies no skip to children living in the same household, and records a separate response for each child, so the two kinds of child enter the average in the same way. Slots corresponding to children a respondent does not have are not applicable and are excluded from the average. A response of “never” is scored 0 rather than treated as missing, so contact reported as absent is retained in the average. Where a response is missing for only some of a respondent’s children, the mean is taken over the remaining children rather than dropping the respondent; where the respondent’s entire self-report questionnaire is missing, the measure is left missing, and the case is handled by complete-case deletion rather than assigned 0. Respondents who have no children are assigned 0, on the grounds that contact with children is then genuinely absent rather than unobserved; this applies to 287 respondents in the analysis sample. Section 4.4 sets out the interpretative limits that follow from this coding.

Interpersonal trust is measured by the item “Do you think most people can be trusted, or is it better to be as careful as possible when dealing with others?” Responses were dichotomized into a binary variable: “better to be as careful as possible” = 0, and “most people can be trusted” = 1, with higher values indicating greater interpersonal trust.

#### 2.3.3. Mediating Variable

The mediating variable is life meaning, measured by a self-assessment item asking respondents to rate “the degree to which you find life meaningful” on an 11-point scale from 0 (very meaningless) to 10 (very meaningful). Higher scores indicate a stronger sense of life meaning.

#### 2.3.4. Moderating Variables

The moderating variables capture two forms of digital social engagement and, in accordance with the research framework, are divided into two dimensions: digital social participation and digital social interaction.

Digital social participation is measured by the item “Over the past year, have you used WeChat?” Responses were dichotomized into a binary variable: “no” = 0, indicating no participation in digital social networks, and “yes” = 1, indicating participation in digital social networks.

Digital social interaction is measured by the item “Over the past year, how often did you share your work or daily life on WeChat Moments?” Responses range from 0 to 6, corresponding to “not applicable/never,” “once every few months,” “once a month,” “2–3 times a month,” “1–2 times a week,” “3–4 times a week,” and “almost every day,” respectively. The category “not applicable” arises from skip logic—respondents who answered “no” to the preceding question on WeChat usage were routed past this item. Therefore, “not applicable” and “never” were merged and assigned a value of 0, indicating no digital social interaction behavior. Higher values indicate more frequent digital social interaction.

#### 2.3.5. Control Variables

To more accurately identify the net effect of social capital on depressive symptoms in older adults, a set of control variables known to influence mental health in older adults was included: age, gender, urban–rural household registration (hukou), marital status, illness/injury severity, and life satisfaction.

Age is measured as the respondent’s chronological age in years. Age is associated with physical decline and social role loss, which may increase depression risk (Poyraz & Bruk Oy, 2025; Shen et al., 2025; Veronese et al., 2017). Gender is a binary variable with female = 0 and male = 1. Gender differences shape psychological resilience and emotional expression patterns, influencing depressive levels (Ching et al., 2016; Jarrad et al., 2025; M. Liu et al., 2024). Urban–rural hukou is a binary variable with rural = 0 and urban = 1. Hukou status captures disparities in healthcare resources, social support, and lifestyle, with documented effects on depression (L. W. Li et al., 2016; Yu et al., 2022). Marital status was entered as a nominal categorical variable with four dummy variables for divorced, widowed, never married and cohabiting respondents, with married respondents as the reference category. Treating the categories as an ordered scale would have imposed an equal-interval assumption that they do not satisfy. Marital status directly affects the availability of emotional support and companionship, serving as an important protective factor against depression (Amegbor et al., 2021; Y. Li et al., 2025). Illness/injury severity is based on the item “How severe do you feel the illness or injury you experienced in the past two weeks is?” and is coded from 0 to 3, representing “not applicable,” “not severe,” “moderate,” and “severe,” respectively (“not applicable” refers to respondents who reported no physical discomfort in the past two weeks and is thus coded as 0). Higher values indicate greater illness severity. Illness/injury severity imposes physical suffering and economic burden and is a well-established risk factor for depression (Yang et al., 2025). Life satisfaction is measured by the item “Are you satisfied with your life?” and is coded from 1 to 5, representing “very dissatisfied” through “very satisfied,” with higher values indicating greater life satisfaction. Life satisfaction reflects an individual’s overall subjective evaluation of quality of life and is closely linked to depression (Koivumaa-Honkanen et al., 2004; Nes et al., 2013). It is included as a covariate because it represents a broad, global evaluation that respondents formed independently of the specific ties and interactions measured here, and it therefore functions as a pre-exposure confounder of the association between social capital and depressive symptoms. The alternative possibility—that life satisfaction lies downstream of social capital and on the pathway to depressive symptoms—would make its inclusion an instance of overadjustment, and Section 3.4.3 accordingly reports the models both with and without it.

### 2.4. Model Specification

To examine the associations between social capital and depressive symptoms in older adults, this study adopts the moderated mediation analytical framework proposed by Wen et al. (2006) and constructs five sequential regression equations in accordance with the theoretical framework and variable specification.

Intergenerational interaction frequency and digital social interaction are ordinal frequency measures and are entered as linear terms; the linearity assumption was examined in sensitivity analyses and is reported in Section 3.4.4.

Equation (1) estimates the total effects of interpersonal trust and intergenerational interaction frequency on depressive symptoms—that is, the overall impact of social capital on mental health.

D_i_ = a_0_ + a_1_T_i_ + a_2_G_i_ + a_3_C_i_ + ε_1i_ (1)

Equation (2) estimates the direct effects of interpersonal trust and intergenerational interaction frequency on life meaning, testing whether cognitive and structural social capital promote life meaning in older adults.

M_i_ = b_0_ + b_1_T_i_ + b_2_G_i_ + b_3_C_i_ + ε_2i_ (2)

Equation (3) includes interpersonal trust, intergenerational interaction frequency, and life meaning simultaneously to test the mediating role of life meaning and the direct effects of social capital on depressive symptoms after controlling for the mediator.

D_i_ = c_0_ + c_1_T_i_ + c_2_G_i_ + c_3_M_i_ + c_4_C_i_ + ε_3i_ (3)

Equation (4) extends Equation (2) by adding digital social interaction and its interaction term with intergenerational interaction frequency, testing whether digital social interaction moderates the relationship between intergenerational interaction frequency and life meaning.

M_i_ = d_0_ + d_1_T_i_ + d_2_G_i_ + d_3_I_i_ + d_4_G_i_ × I_i_ + d_5_C_i_ + ε_4i_ (4)

Equation (5) extends Equation (3) by adding digital social participation and its interaction term with interpersonal trust, testing whether digital social participation moderates the relationship between interpersonal trust and depressive symptoms.

D_i_ = e_0_ + e_1_T_i_ + e_2_G_i_ + e_3_M_i_ + e_4_P_i_ + e_5_T_i_ × P_i_ + e_6_C_i_ + ε_5i_ (5)

In the above equations, D_i_ denotes the depressive symptom score of older adult i; T_i_ and G_i_ denote interpersonal trust and intergenerational interaction frequency, respectively; M_i_ denotes life meaning; P_i_ and I_i_ denote digital social participation and digital social interaction, respectively; and C_i_ represents the vector of control variables including age, gender, urban–rural hukou, marital status, illness/injury severity, and life satisfaction. a_0_, b_0_, c_0_, d_0_, and e_0_ are the intercepts, and ε denotes the random error term.

Mediation was evaluated from the indirect association and its confidence interval rather than from the significance of the total effect or from the attenuation of the direct coefficient. The indirect association through life meaning was computed as the product of the relevant coefficient in Equation (2) and the coefficient on life meaning in Equation (3), and its confidence interval was obtained both by the design-based delta method and by bootstrap resampling of primary sampling units. An indirect association was taken as supported when its confidence interval excluded zero, irrespective of whether the total effect or the remaining direct association was itself significant, and the proportion of the total association accounted for by the indirect path is reported alongside. The significance of the total effect is therefore not treated as a precondition for mediation, and no distinction between partial and full mediation is drawn from the residual direct coefficient.

The two moderating hypotheses occupy different positions in the model and are evaluated accordingly. Digital social interaction is expected to moderate the a-path of the indirect pathway, so Equation (4) is used to estimate conditional indirect associations—the indirect association evaluated at specified values of digital social interaction—together with the index of moderated mediation, which is the product of the interaction coefficient in Equation (4) and the coefficient on life meaning in Equation (3). The index tests directly whether the indirect association is conditional, and its confidence interval is obtained by the same two methods. Digital social participation, by contrast, is expected to moderate the direct association between interpersonal trust and depressive symptoms and is therefore evaluated from the interaction coefficient in Equation (5) and from the association between interpersonal trust and depressive symptoms at each value of participation. The distinction matters for terminology: only the first of the two constitutes moderated mediation, and the framework is described as a moderated mediation model on that basis alone. The interaction terms in Equations (4) and (5) are tested directly, from the interaction coefficients themselves and from the conditional estimates and are not made conditional on the significance of the main effects in those equations. Where an association is reported at a particular value of a moderator, that value is an actual point on the scale rather than a hypothetical level, the estimate is the fitted association at that value with all other covariates held at their sample means, and it is reported with its confidence interval. The figures display predicted values obtained in the same way from the fitted equations so that the presented lines and the reported numbers are the same quantities.

To mitigate structural multicollinearity between the main-effect terms and the product terms, the four variables entering the interaction terms—interpersonal trust, intergenerational interaction frequency, digital social participation, and digital social interaction—were mean-centered before the products were formed. Two of the four—interpersonal trust and digital social participation—are dichotomous rather than continuous: interpersonal trust is coded 1 for respondents who believe that most people can be trusted and 0 for those who believe it is better to be careful, and digital social participation is coded 1 for WeChat users and 0 for non-users. Mean-centering a dichotomous variable recenters it on zero, which changes the intercept and the interpretation of the accompanying main-effect coefficients, but it does not treat the variable as continuous. Each interaction term is the product of two mean-centered variables: digital social interaction multiplied by intergenerational interaction frequency, which enters Equation (4), and digital social participation multiplied by interpersonal trust, which enters Equation (5). The equations retain these variables in their original metric; because a mean-centered variable differs from its original only by a constant, this yields the same fitted model and the same interaction coefficients and conditional estimates as a specification in which every term is mean-centered.

All models were estimated by survey-weighted least squares or survey-weighted logistic regression using the sampling design described in Section 2.2. Total, direct and indirect associations were estimated from the same fitted equations, and their standard errors and confidence intervals were obtained both by the design-based delta method and by 5000 bootstrap replications resampling primary sampling units; the two agreed to two decimal places. The index of moderated mediation and the conditional indirect associations were obtained in the same way.

## 3. Results

### 3.1. Sample Characteristics and Descriptive Statistics

The sociodemographic profile of the weighted sample was as follows. The majority were adults aged 60–69 years (2772/4394, 61.57%), followed by those aged 70–79 years (1422/4394, 33.46%); adults aged 80 years and above accounted for a smaller proportion (200/4394, 4.97%). Gender was relatively balanced (male: 2286/4394, 52.40%). Urban respondents accounted for 54.71% of the weighted sample (2175/4394) and rural respondents for 45.29% (2219/4394). The majority were married with a spouse (3663/4394, 83.57%). Over 60% reported no illness or injury (2776/4394, 64.89%), while 564 respondents (11.62% weighted) reported severe conditions. Life satisfaction was high, with 79.96% reporting being satisfied or very satisfied. Percentages are weighted for the sampling design, and counts are unweighted. Because complete-case selection excluded approximately one third of the eligible sample, these characteristics should not be read as nationally representative of all Chinese older adults.

Table 1 also reports the descriptive statistics for the study variables. The mean depressive symptom score was 13.65 (SD 4.48, range 8–32), and the mean life meaning score was 7.66 (SD 2.16, range 0–10).

The mean intergenerational interaction frequency was 3.12 (SD 1.88, range 0–6). The majority of older adults (2343/4394, 54.54%) reported that most people can be trusted, while 2051 (45.46%) reported that most people cannot be trusted.

Fewer than half of the respondents (2058/4394, 45.66%) reported having used WeChat in the past year. The vast majority (3516/4394, 80.26%) reported never sharing on WeChat Moments, and 132 respondents (2.98%) reported sharing almost every day.

### 3.2. Bivariate Correlations

Pearson correlations among all variables are reported in Table 2. Both intergenerational interaction frequency and interpersonal trust were significantly negatively correlated with depressive symptoms. The mediating variable, life meaning, was significantly negatively correlated with depressive symptoms. Furthermore, both intergenerational interaction frequency and interpersonal trust were significantly positively correlated with life meaning. Both digital social participation and digital social interaction were significantly negatively correlated with depressive symptoms. Additionally, intergenerational interaction frequency was significantly positively correlated with both digital social participation and digital social interaction.

### 3.3. Regression Results and Hypothesis Testing

Model diagnostics were conducted before the models were interpreted. Multicollinearity was assessed separately for each of the five models rather than for a single pooled specification: Table 3 reports the variance inflation factors for every term in each model; no variance inflation factor exceeds 1.24, and the mean variance inflation factor ranges from 1.05 to 1.10 across the five models, well below the conventional threshold of 10. The highest values are those of life meaning and life satisfaction, whose conceptual overlap would be expected to produce them. Heteroscedasticity was assessed in the same step. The White test was applied to each of the five models and was significant in every case (chi-squared = 199.57 with 64 degrees of freedom for Model 1, 264.40 with 64 for Model 2, 246.12 with 77 for Model 3, 294.34 with 89 for Model 4, and 281.97 with 101 for Model 5; *p* < 0.001 throughout). Heteroscedasticity affects the conventional standard errors and the inference based on them—t tests, *p* values and confidence intervals—rather than the OLS coefficients themselves: re-estimating the first model with conventional and with heteroscedasticity-robust standard errors leaves the coefficients numerically identical and changes the standard errors by no more than 2%. Conventional standard errors are therefore not used for inference here. As set out in Section 2.4, the five models are estimated under the CFPS sampling design, so their standard errors are design-based and the confidence intervals reported alongside them come from bootstrap replications that resample primary sampling units rather than from an assumption of homoscedasticity. These diagnostics, like the descriptive statistics, are computed without sampling weights and therefore describe the properties of the data rather than the precision of the survey-weighted estimates. The regression results are presented in Table 4.

Model 1 examined the direct effects of interpersonal trust and intergenerational interaction frequency on depressive symptoms. Both interpersonal trust (β = −0.756, *p* < 0.001) and intergenerational interaction frequency (β = −0.238, *p* < 0.001) were significantly negatively associated with depressive symptoms, supporting Hypotheses H1a and H1b.

Model 2, with life meaning as the dependent variable, showed that interpersonal trust (β = 0.323, *p* < 0.001) and intergenerational interaction frequency (β = 0.093, *p* < 0.001) were both significantly positively associated with life meaning. These findings indicate that higher interpersonal trust and more frequent intergenerational interaction were associated with a stronger sense of life meaning in older adults.

Model 3 added life meaning to the specification of Model 1. Life meaning was negatively associated with depressive symptoms (β = −0.505, *p* < 0.001), supporting Hypothesis H3. With life meaning in the model, interpersonal trust (β = −0.592, *p* < 0.001) and intergenerational interaction frequency (β = −0.191, *p* < 0.001) both remained negatively associated with depressive symptoms, although the absolute magnitudes of both coefficients were smaller than in Model 1. These estimates are reported for completeness; the criterion used to evaluate mediation is the indirect association and its confidence interval, which are reported below and in Table 5.

For the path from interpersonal trust to depressive symptoms through life meaning, the indirect association was −0.163 (95% CI −0.252 to −0.074) and the direct association was −0.592 (95% CI −0.911 to −0.274); neither interval contained zero, and the indirect path accounted for 21.6% (95% CI 7.8% to 35.4%) of the total association. For the path from intergenerational interaction frequency through life meaning, the indirect association was −0.047 (95% CI −0.072 to −0.021) and the direct association was −0.191 (95% CI −0.270 to −0.112), with 19.6% (95% CI 9.3% to 30.0%) of the total association accounted for by the indirect path. Because both the indirect and the direct associations are non-zero, life meaning accounts for part rather than all of the association between each social capital indicator and depressive symptoms (H2a, H2b). Confidence intervals were obtained by the design-based delta method and by 5000 bootstrap replications resampling primary sampling units, and the two agreed to two decimal places. The total, direct and indirect associations reported above are estimated from the same fitted equations as the coefficients in Table 4, the indirect association being the product of the corresponding coefficients in Models 2 and 3; the two tables therefore report one specification rather than two.

**Figure 2:**
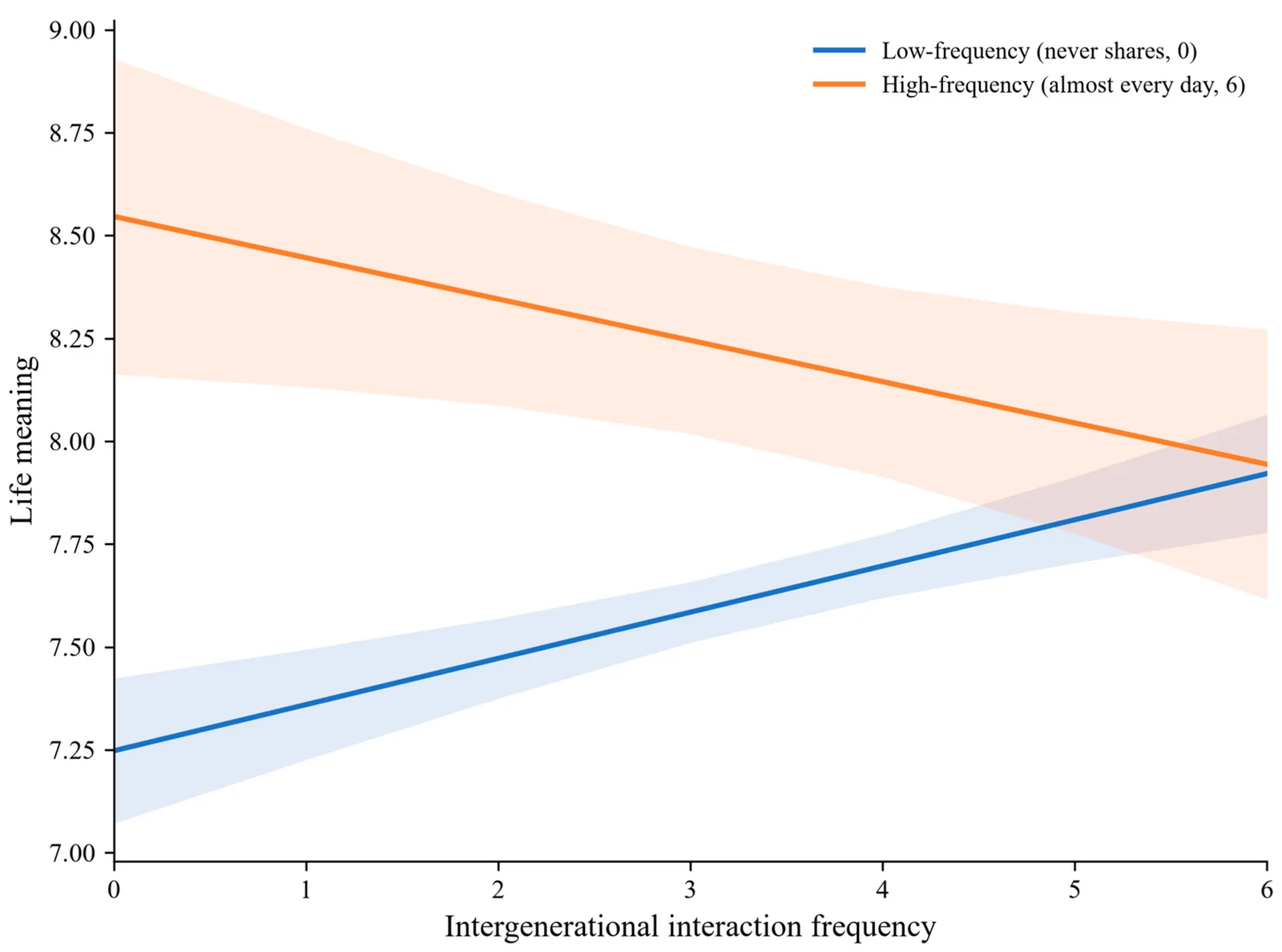
Association between intergenerational interaction frequency and life meaning at low and high levels of digital social interaction. The lines are predicted values from the fitted equation (Model 4), with all other covariates held at their sample means; they are not separate fits within groups. Low-frequency denotes a score of 0 on digital social interaction (never shares content on WeChat Moments), reported by 80.3% of the weighted analysis sample, and high-frequency denotes a score of 6 (almost every day), reported by 3.0%. The fitted slope of intergenerational interaction frequency on life meaning is 0.112 (95% CI 0.065 to 0.160) at a score of 0 and −0.100 (95% CI −0.192 to −0.009) at a score of 6.

Model 4, with life meaning as the dependent variable, examined whether digital social interaction moderates the relationship between intergenerational interaction frequency and life meaning. The interaction term (intergenerational interaction frequency × digital social interaction) was significant and negative (β = −0.035, *p* < 0.001). Because the interaction is negative and the main effect of intergenerational interaction frequency on life meaning is positive, the fitted association between intergenerational interaction frequency and life meaning declines across the range of digital social interaction: it was 0.112 (95% CI 0.065 to 0.160) at a score of 0, 0.077 (95% CI 0.036 to 0.118) at a score of 1, 0.006 (95% CI −0.044 to 0.056) at a score of 3, and −0.100 (95% CI −0.192 to −0.009) at a score of 6. Thus, the association is positive and clearly distinguishable from zero at the lowest value of the scale, indistinguishable from zero in the middle of the scale, and negative in point estimate at the highest value. That highest value is reported by only 2.98% of the weighted analysis sample, however, and the estimate at it rests on a linear extrapolation at the edge of the scale rather than on a well-populated level of the moderator; the moderation is therefore described here as a weakening of the positive association rather than as a reversal of it. The pattern does not depend on where the moderator is divided: entering it as a binary indicator at any cut point from 1 to 5, the interaction remains negative, and its confidence interval excludes zero (coefficients between −0.11 and −0.16). The moderating effect is illustrated in Figure 2.

Because digital social interaction moderates the a-path of the indirect pathway, the moderated mediation can be evaluated directly rather than inferred from the interaction coefficient. The indirect association through life meaning was −0.057 (95% CI −0.088 to −0.030) among respondents who never shared on WeChat Moments, −0.039 (95% CI −0.065 to −0.017) at once every few months, and −0.003 (95% CI −0.031 to 0.021) at two to three times a month; it was −0.072 (95% CI −0.109 to −0.038) one standard deviation below the mean of digital social interaction and −0.019 (95% CI −0.044 to 0.001) one standard deviation above it. The second pair of values is reported for comparability with conventional practice rather than because it describes an attainable level of the moderator: one standard deviation below the mean of digital social interaction is −0.84, whereas the scale begins at 0, so that value lies outside the range of the measure and the association estimated there is an extrapolation. The indirect association therefore weakens as digital social interaction increases, and by the higher frequencies its confidence interval includes zero. The index of moderated mediation, which tests directly whether the indirect association is conditional, was 0.018 (95% CI 0.008 to 0.029). Because that interval excludes zero, the indirect pathway through life meaning is conditional on digital social interaction, which is what H4b proposes; these estimates are reported in Panel B of Table 5. The moderation estimated in Model 5 concerns the direct association between interpersonal trust and depressive symptoms and is not itself a moderated mediation effect; it is reported separately in Panel C.

**Figure 3:**
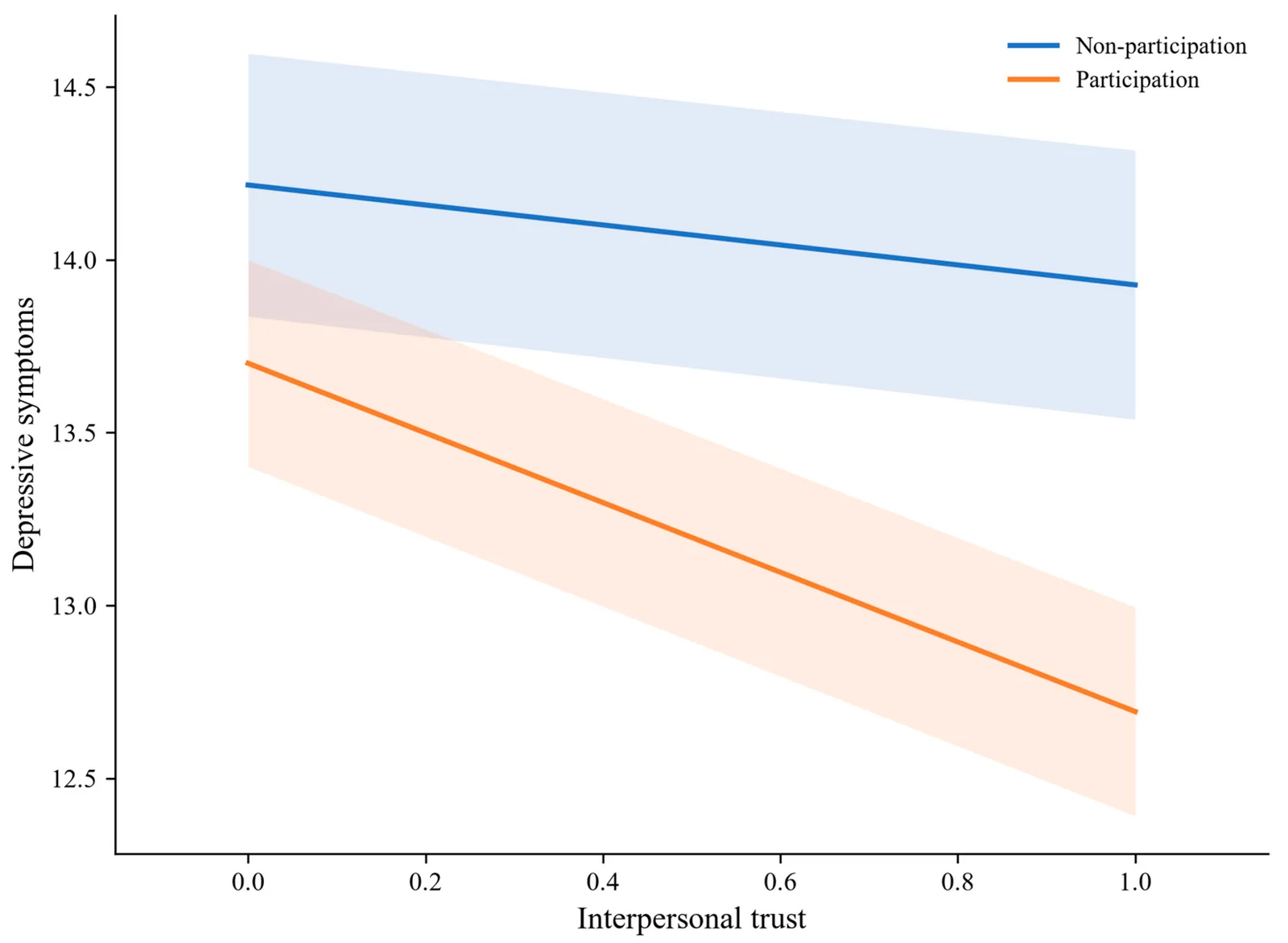
Association between interpersonal trust and depressive symptoms among respondents who did and did not participate in digital social networks. The lines are predicted values from the fitted equation (Model 5), with all other covariates held at their sample means; they are not separate fits within groups. The fitted slope of interpersonal trust on depressive symptoms is −1.008 (95% CI −1.390 to −0.625) among participants and −0.289 (95% CI −0.732 to 0.154) among non-participants.

Model 5, with depressive symptoms as the dependent variable, examined whether digital social participation moderates the relationship between interpersonal trust and depressive symptoms. The interaction term (interpersonal trust × digital social participation) was significant and negative (β = −0.719, *p* = 0.011). Because participation is dichotomous, the interaction is the difference between the two fitted associations: the association between interpersonal trust and depressive symptoms was −1.008 (95% CI −1.390 to −0.625) among respondents who used WeChat and −0.289 (95% CI −0.732 to 0.154) among those who did not. The association is therefore negative and distinguishable from zero among participants, and not distinguishable from zero among non-participants; it is the difference between the two—the interaction term—that constitutes the test of Hypothesis H4a. The moderating effect is illustrated in Figure 3.

### 3.4. Robustness Checks

#### 3.4.1. Alternative Model Specification: Logit Model

The continuous dependent variables were additionally transformed into binary outcomes—depressive symptoms were recoded as “depressed” or “not depressed” and life meaning as “life is meaningful” or “life is not meaningful”—and the five models were re-estimated using logistic regression with robust standard errors. Depressive symptoms were dichotomised at CES-D-8 ≥ 16. The CES-D-8 sums eight items scored 1 to 4, so scores range from 8 to 32 in this sample, and the cutoff of 16 accordingly lies at one third of the scale range rather than at a scale midpoint. Life meaning (0–10) was dichotomised at ≥ 6. We note that 6 is not the midpoint of a 0–10 scale—the midpoint is 5—and we therefore re-estimated every model at ≥5 as well; the pattern of coefficients and their significance was unchanged, so the results do not depend on the particular threshold adopted. Dichotomisation necessarily discards information and reduces statistical efficiency relative to the continuous specification. The continuous models in Section 3.3 and Section 3.4 are therefore the primary specification, and this analysis is presented as a supplementary check on the direction and significance of the associations rather than as an independent test of robustness.

Under the logit specification, the direction and significance of the core coefficients were consistent with the continuous models, with detailed results presented in Table 6. In Models 1 and 2, both interpersonal trust and intergenerational interaction frequency were associated with a lower probability of being above the depression cutoff and with a higher probability of perceiving life as meaningful (*p* < 0.01). In Model 3, being above the life-meaning cutoff was associated with a lower probability of being above the depression cutoff (*p* < 0.01), and the absolute coefficients on interpersonal trust and intergenerational interaction frequency both decreased relative to Model 1, which is the pattern expected if life meaning carries part of the association; the criterion used to evaluate mediation remains the indirect association reported in Table 5. In Model 4, the interaction between intergenerational interaction frequency and digital social interaction was negative and significant (*p* < 0.05), which is weaker evidence than in the continuous specification and is reported as such. In Model 5, the interaction between interpersonal trust and digital social participation was negative and significant (*p* < 0.05). Across the five models, 1445 respondents (31.62% unweighted, 29.55% weighted) were above the CES-D-8 cutoff and 3577 (78.27% unweighted, 79.13% weighted) were at or above the life-meaning cutoff.

#### 3.4.2. Winsorization

All continuous variables were winsorised at the 1st and 99th percentiles, and the five baseline models were re-estimated. Winsorisation is not equally informative for all variables, and the difference is worth stating. Several variables are measured on naturally bounded scales whose extreme values are legitimate scale points rather than outliers: intergenerational interaction frequency and digital social interaction are bounded at 0 and 6, and their 1st and 99th percentiles coincide with the endpoints, so winsorisation replaces no observations for either variable (0 of 4570 in both cases). Life meaning (0–10) and life satisfaction (1–5) are similarly bounded, and winsorisation replaced only 33 and 32 observations respectively (0.72% and 0.70%). Depressive symptoms (8–32) is the one variable with a gap between its 99th percentile (27) and its maximum (32), and there, winsorisation replaced 37 observations (0.81%). Rather than treating the unchanged direction and significance of the coefficients as sufficient evidence of robustness, which it is not, we report influence diagnostics for the baseline depression model. Cook’s distance exceeded 4/n for 227 observations, leverage exceeded 2k/n for 143, the absolute standardized residual exceeded 3 for 42, and the absolute DFBETA for interpersonal trust exceeded 2/√n for 220. Re-estimating the baseline model after dropping the observations in the top 1% of Cook’s distance (*N* = 4525) left the coefficients on interpersonal trust (−0.661) and intergenerational interaction frequency (−0.166) essentially unchanged, with the same direction and significance. This re-estimation, like the diagnostics and the replacement counts above, is computed without sampling weights and describes the properties of the data rather than the survey-weighted estimates reported in Table 4. Extreme values are therefore influential in a small number of cases but do not drive the reported associations.

#### 3.4.3. Sensitivity to the Inclusion of Life Satisfaction

Because life satisfaction is strongly associated with both life meaning and depressive symptoms, its inclusion among the control variables could either remove confounding or, if it lies on the pathway from social capital to depressive symptoms, induce overadjustment. The models were therefore re-estimated with and without it. These comparisons use unweighted estimates, because the question here concerns the covariate specification rather than the sampling design; unweighted and survey-weighted estimates were already shown to agree in direction and significance (Section 2.2 and Section 4.4). They should therefore be compared with one another rather than with the survey-weighted coefficients in Table 5. Excluding life satisfaction leaves the direction and significance of every focal coefficient unchanged, but it does move some of them appreciably: the association between interpersonal trust and depressive symptoms increases from −0.67 to −0.84, the association between interpersonal trust and life meaning from 0.20 to 0.37, and the association between intergenerational interaction frequency and life meaning from 0.09 to 0.10, while the association between intergenerational interaction frequency and depressive symptoms moves only from −0.17 to −0.18 and that between life meaning and depressive symptoms is essentially unaffected. The sensitivity of the trust coefficients to this choice reflects the conceptual overlap between trust and global life evaluation, and the version adjusted for life satisfaction is retained as the primary specification because, on theoretical grounds, life satisfaction is a pre-exposure evaluation rather than a consequence of the specific ties and interactions measured here. The unadjusted estimates are reported here so that readers can assess the magnitude of the adjustment.

#### 3.4.4. Linearity of the Ordinal Frequency Measures

Intergenerational interaction frequency and digital social interaction are measured on ordinal frequency scales, and the models enter both as linear terms. Two complementary checks were carried out on that assumption. First, a quadratic term was added to each equation in which the variable appears; the coefficient on the squared term was not significant when intergenerational interaction frequency predicted life meaning (*p* = 0.697) or depressive symptoms (*p* = 0.780), nor when digital social interaction predicted life meaning (*p* = 0.935) or depressive symptoms (*p* = 0.123), so a linear specification is not rejected in any of the four cases. Second, each variable was entered categorically, and the joint test of the category coefficients was significant in every case (*p* = 0.003 for intergenerational interaction frequency predicting life meaning, *p* < 0.001 in the other three), while the category coefficients increased monotonically across the scale rather than reversing direction, which is the pattern a non-linear relationship would produce. The linear specification is therefore retained.

## 4. Discussion

### 4.1. Principal Findings

This study examined the associations between social capital and depressive symptoms in older adults using a moderated mediation framework. Based on the CES-D-8, interpersonal trust, intergenerational interaction frequency, life meaning, and digital engagement measures from the 2022 CFPS data, all seven hypotheses were supported. The core findings are threefold.

First, both structural social capital (intergenerational interaction frequency) and cognitive social capital (interpersonal trust) were negatively associated with depressive symptoms. This finding aligns with the broader literature on the positive association between social support and mental health (Z. Xu et al., 2022; Zhou et al., 2022) and extends it within the Chinese older adult population using nationally representative data. The contribution of this study, however, lies not in documenting this established association but in examining whether its strength differs between older adults who engage digitally and those who do not: where the offline domain and a digitally mediated social space meet, the strength of the association between social capital and depressive symptoms is not fixed.

Second, life meaning accounted for part of the association between both dimensions of social capital and depressive symptoms. Whether through intergenerational interaction frequency (objective network ties) or interpersonal trust (subjective psychological disposition), the association between social capital and depressive symptoms is partly accounted for by older adults’ sense that their lives are meaningful. This finding suggests that the value of social capital lies not only in providing external support to buffer the impact of stressful events but also in helping older adults reconstruct an internal sense of self-worth and existential purpose in the later stages of the life course.

From a practical standpoint, these results point to two potential targets for future intervention research rather than to recommendations. The first concerns the social resources available to older adults: because the associations we observe run through life meaning as well as through social capital itself, interventions that sustain meaningful social activity in later life are worth testing, although the present cross-sectional design cannot establish that such interventions would reduce depressive symptoms. The second concerns digital social engagement, which our results suggest is not uniformly beneficial, since participation and frequency of sharing were associated with different patterns in the models we estimated. Both remain untested here: no intervention of any kind was evaluated in this study, and we therefore present them as directions for future research rather than as implications of our findings.

### 4.2. The Asymmetric Moderating Effects of Digital Social Engagement

With life meaning included as the mediator of interest, the moderating effects of digital social engagement can be interpreted within a clear explanatory framework. The core finding—that the two forms of digital social engagement moderated two distinct pathways in opposite directions—is, we believe, the most interesting result of this study. We propose that this asymmetry reflects a difference in the process each indicator bears on rather than a difference in the type of digital inequality it represents. Digital social participation bears on the interpersonal-trust pathway, which concerns how a disposition toward others is expressed in social relations and how that bears on affect; digital social interaction bears on the intergenerational-interaction pathway, which concerns the maintenance of meaning through ongoing social activity. Under this reading the two indicators need not act in the same direction, because the processes to which they are relevant differ. We emphasize that this is the interpretation we propose, not a property of the indicators that our data establish: neither process was measured directly, and the alignment is a theoretical proposition advanced here.

This finding has a theoretical implication that we state tentatively. It suggests that digital social engagement should be treated not only as an antecedent of mental health but also as a contextual condition under which the associations of social capital differ, which is the framing our two interaction terms were specified to test. Digital social engagement is, in this sense, not merely a technological gap to be bridged but a feature of the social context in which older adults’ social relations unfold. Whether this framing survives replication and whether the two indicators should be read as bearing on different processes are questions for further research.

If, as we propose, the two indicators bear on different processes, the pattern is that the negative association between interpersonal trust and depressive symptoms was stronger among older adults who participated in digital social networks. We can only speculate about what might produce such a pattern, and we do so explicitly as speculation: greater familiarity with the technology may allow older adults who are already disposed to trust others to act on that disposition in an additional social setting. The variables needed to examine this—perceived psychological barriers to using digital technology, cognitive accessibility, and perceived social support—were not measured in the present study, and measures that could follow from this line of reasoning, such as age-friendly device design or community-based digital-skills provision, have not been evaluated. We therefore present them as hypotheses for future research rather than as implications of our findings.

In the behavioral dimension, the positive association between intergenerational interaction frequency and life meaning was weaker among older adults who shared more frequently online. One possible explanation is substitution: sharing online and interacting with one’s children both involve time and emotional investment, and if the two compete for a fixed amount of either, the association between intergenerational interaction and meaning could weaken as sharing increases. This is a possible explanation rather than an established one, since we did not measure how respondents allocate time between online and offline contact, the quality of their face-to-face interaction, or their emotional investment in either. We therefore do not conclude that online activity displaces offline relationships, and we frame the balance between the two as a question for future longitudinal work rather than as a recommendation.

### 4.3. Comparison with Prior Work

The findings of this study extend the existing literature in three key respects.

First, prior research on older adults’ mental health has largely examined social capital (Z. Xu et al., 2022; Zhou et al., 2022) and the digital divide as parallel, independent determinants, without integrating them into a unified analytical framework that captures their interaction. This study departs from this siloed approach by constructing an integrated moderated mediation framework in which digital social engagement functions as a contextual condition that moderates—rather than simply coexists with—the associations of social capital.

Second, prior research has not systematically distinguished between different forms of digital social engagement when examining their moderating effects on the social capital–mental health relationship. The present findings indicate that the moderating effects of digital social engagement are form-specific and directionally opposite. The negative association between interpersonal trust and depressive symptoms was stronger among older adults who participated in digital social networks. In contrast, the positive association between intergenerational interaction frequency and life meaning was weaker among older adults with more frequent digital social interaction. This asymmetry, which we propose reflects an alignment between each indicator and a specific dimension of social capital, calls into question any undifferentiated characterization of the moderating role of digital social engagement.

Third, while prior research has documented the negative associations between social capital and depressive symptoms through external support pathways such as stress buffering (Bovier et al., 2004; Cohen & Wills, 1985), the internal psychological pathway through which social capital is associated with mental health has received less attention. This study provides empirical evidence that life meaning accounts for part of the association between both structural and cognitive social capital and depressive symptoms. This finding aligns with perspectives from logotherapy (Botîlcă, 2024; Russo-Netzer & Ameli, 2021) and social identity theory (Spears, 2021; Tajfel & Turner, 2000) but extends them by demonstrating the mediating role of life meaning within a large, nationally representative sample of older adults.

It is worth stating explicitly what life meaning adds here, given that several psychological mediators of the social-relationship–depression association are already well established. Loneliness, perceived social support and self-esteem describe the availability and perceived quality of social resources, and life satisfaction is a global evaluative judgement about one’s life. Meaning in life is narrower: it concerns whether one’s existence is experienced as coherent and purposeful (H. M. Tian et al., 2025). Our results suggest that this pathway is not uniform across older adults. The association between intergenerational interaction frequency and life meaning weakened as digital social interaction increased, whereas digital social participation strengthened the negative association between interpersonal trust and depression. The psychological pathway linking social relationships to mental health therefore appears conditional on the digital context in which older adults’ social lives unfold—a condition against which the mediators listed above have not been tested. This extends social capital theory in later life by showing that the returns to structural and cognitive social capital are not fixed properties of the capital itself but depend on the digital channels through which older adults sustain their social lives. One qualification bears directly on this pathway: life meaning was measured with a single item, and a single item cannot separate measurement error from true variation in the construct. The indirect association through life meaning is therefore best read as approximate rather than as a precise estimate of the pathway, which is the pathway on which the conditional pattern described above rests.

### 4.4. Limitations

Several limitations of this study should be acknowledged. First, the cross-sectional design precludes causal inference. Although the moderated mediation framework specifies directional pathways grounded in established theory, longitudinal or experimental designs are needed to establish temporal precedence and rule out reverse causality. Future research employing panel data across multiple CFPS waves would be valuable for validating the proposed pathways over time.

Second, all measures were derived from self-reported survey items. Depressive symptoms were assessed using the CES-D-8 rather than clinical diagnostic instruments, and life meaning was captured by a single-item measure. While the CES-D-8 is a well-validated screening tool, the mediating variable rests on a single item, and a single item cannot separate measurement error from true variation in life meaning. The indirect association through life meaning is therefore best read as approximate rather than as a precise estimate of the pathway, and the same qualification applies to the proportion of the total association it accounts for. Future studies would benefit from multi-item scales of life meaning and from clinical assessments of depressive symptoms.

Third, digital social participation was measured solely through WeChat usage, and digital social interaction was measured solely through WeChat Moments sharing. Although WeChat is the dominant social media platform among older adults in China and the only digital social platform measured in CFPS 2022, this measurement may not fully capture the diversity of older adults’ digital social engagement, such as participation in short-video platforms, online communities, or other social networking applications. Non-use of WeChat therefore indicates non-use of that platform only; it should not be read as exclusion from digital social life, and frequency of sharing on WeChat Moments does not describe the full range of online activities in which a respondent may engage.

A further measurement limitation concerns intergenerational interaction frequency. Because respondents who have no children are assigned a frequency of zero, the measure cannot distinguish them from respondents who have children but never see them: the two are coded identically even though the underlying situations differ, and for a respondent without children, the item does not record a level of intergenerational contact at all. The measure also does not record living arrangements, so co-residence with a child is not separately identified and the same reported frequency can arise from a child living in the same household or from a child living elsewhere whom the respondent sees equally often.

Finally, although the analyses are weighted for the CFPS sampling design, they rest on the 4394 respondents who had complete data on all study variables and a valid sampling weight. Complete-case selection excluded approximately one third of the eligible sample (2290 of 6860), and weighting cannot correct for this. The comparison reported in Section 2.2 shows that the excluded respondents were markedly older and less digitally engaged, so the analysis sample is not a random subset of eligible older adults even after weighting. A multiple-imputation sensitivity analysis that recovers the excluded cases leaves the focal coefficients essentially unchanged, but its high fraction of missing information means that it corroborates rather than removes this limitation. The weighted estimates should therefore be read as descriptive of the population the weights represent conditional on that selection; unweighted estimates were examined as a robustness check and were similar in direction and significance.

Fourth, the findings are based on a Chinese national sample. While China offers a particularly relevant context for studying the intersection of rapid aging and rapid digitization, the generalizability of the asymmetric moderation pattern to other cultural and institutional settings remains to be established.

## 5. Conclusions

This study examined whether two specific indicators of social capital—intergenerational interaction frequency and interpersonal trust—are associated with depressive symptoms among older adults in China, whether life meaning carries part of those associations, and whether two forms of digital social engagement moderate them. Both indicators were negatively associated with depressive symptoms (intergenerational interaction frequency: β = −0.238, *p* < 0.001; interpersonal trust: β = −0.756, *p* < 0.001) and positively associated with life meaning, and the indirect associations through life meaning were −0.047 (95% CI of −0.072 to −0.021) and −0.163 (95% CI of −0.252 to −0.074), accounting for 19.6% and 21.6% of the corresponding total associations. The two digital measures moderated different associations and in opposite directions: the negative association between interpersonal trust and depressive symptoms was stronger among respondents who used WeChat than among those who did not (interaction −0.719, *p* = 0.011), whereas the positive association between intergenerational interaction frequency and life meaning weakened as sharing on WeChat Moments became more frequent (interaction −0.035, *p* < 0.001; index of moderated mediation 0.018, 95% CI 0.008 to 0.029). Because the design is cross-sectional, these estimates describe associations observed at a single time point; they are consistent with the proposed model but do not establish the ordering it implies.

Read together, the results place digital social engagement as a condition under which the associations between specific social capital indicators and mental health differ, rather than as an independent determinant of mental health. Two directions for future work follow. The asymmetry between the two digital measures was specified before the analysis on theoretical grounds, and comparing it with other plausible pairings requires measures of the psychological acceptance of technology and of the content of online interaction that the CFPS does not provide. More fundamentally, a cross-sectional design cannot separate the temporal ordering implied by the mediation model from its reverse, so longitudinal or intervention studies are needed before these associations can be read as a process. Accordingly, the practical implications are offered as potential targets rather than as recommendations: digital participation might warrant support where interpersonal trust is already present, and intensive online sharing might warrant attention where the maintenance of meaning through family contact is the concern. By connecting the social capital, digital divide and mental health literatures, the study offers a specification for such work to test rather than a conclusion for it to rely on.

## Figures and Tables

**Table 1:** Descriptive statistics of study variables (*N* = 4394).

Variable	Value
Depressive symptoms, mean (SD)	13.65 (4.48)
Interpersonal trust, *n* (weighted %)	
Distrustful of others	2051 (45.46)
Trustful of others	2343 (54.54)
Intergenerational interaction frequency, mean (SD)	3.12 (1.88)
Life meaning, mean (SD)	7.66 (2.16)
Digital social participation, *n* (weighted %)	
Non-participation	2336 (54.34)
Participation	2058 (45.66)
Digital social interaction, *n* (weighted %)	
Never	3516 (80.26)
Once every few months	212 (5.09)
Once a month	145 (2.98)
2–3 times a month	147 (3.65)
1–2 times a week	177 (3.75)
3–4 times a week	65 (1.29)
Almost every day	132 (2.98)
Age, *n* (weighted %)	
60–69	2772 (61.57)
70–79	1422 (33.46)
80–89	190 (4.71)
90–99	10 (0.26)
Gender, *n* (weighted %)	
Female	2108 (47.60)
Male	2286 (52.40)
Hukou, *n* (weighted %)	
Rural	2219 (45.29)
Urban	2175 (54.71)
Marital status, *n* (weighted %)	
Divorced	76 (1.87)
Widowed	607 (13.45)
Never married	27 (0.62)
Cohabiting	21 (0.49)
Married (with spouse)	3663 (83.57)
Illness/injury severity, *n* (weighted %)	
None	2776 (64.89)
Not severe	268 (6.67)
Moderate	786 (16.81)
Severe	564 (11.62)
Life satisfaction, *n* (weighted %)	
Very dissatisfied	31 (0.58)
Dissatisfied	100 (2.05)
Fairly satisfied	801 (17.40)
Satisfied	1392 (31.61)
Very satisfied	2070 (48.35)

Note. *N* = 4394 respondents with valid CFPS sampling weights. Counts are unweighted; percentages are weighted for the sampling design. Standard deviations are design-based. Married respondents are the reference category in the regression models.

**Table 2:** Pearson correlation matrix of study variables.

	(1)	(2)	(3)	(4)	(5)	(6)
Depressive symptoms	1.000					
Interpersonal trust	−0.134 ***	1.000				
Intergenerational interaction frequency	−0.120 ***	0.030 **	1.000			
Life meaning	−0.348 ***	0.105 ***	0.107 ***	1.000		
Digital social participation	−0.117 ***	−0.039 ***	0.046 ***	0.006	1.000	
Digital social interaction	−0.122 ***	0.014	0.083 ***	0.056 ***	0.455 ***	1.000

Note: Correlations are computed without sampling weights and describe the bivariate associations among the study variables. *** *p* < 0.01, ** *p* < 0.05.

**Table 3:** Variance inflation factors for each of the five regression models.

Variable	Model 1	Model 2	Model 3	Model 4	Model 5
Interpersonal trust	1.03	1.03	1.04	1.03	1.04
Intergenerational interaction frequency	1.07	1.07	1.08	1.08	1.08
Life meaning			1.24		1.24
Digital social participation					1.18
Digital social interaction				1.10	
Intergenerational interaction frequency × digital social interaction				1.04	
Interpersonal trust × digital social participation					1.00
Age	1.11	1.11	1.11	1.12	1.22
Gender	1.06	1.06	1.06	1.06	1.06
Hukou	1.07	1.07	1.07	1.10	1.12
Marital status (ref. married)					
Divorced	1.02	1.02	1.02	1.02	1.02
Widowed	1.12	1.12	1.12	1.12	1.12
Never married	1.02	1.02	1.02	1.02	1.02
Cohabiting	1.00	1.00	1.00	1.00	1.00
Illness/injury severity	1.05	1.05	1.06	1.05	1.06
Life satisfaction	1.04	1.04	1.22	1.04	1.23
Mean VIF	1.05	1.05	1.09	1.06	1.10

Note: Models 1–5 correspond to the columns of Table 4. Variance inflation factors are computed from the unweighted specification of each model, because they describe the collinearity of the design matrix rather than the precision of the survey-weighted estimates. A dash indicates that the term is not included in that model.

**Table 4:** Survey-weighted multiple linear regression results (*N* = 4394).

	Model 1	Model 2	Model 3	Model 4	Model 5
Variable	Depressive Symptoms	Life Meaning	Depressive Symptoms	Life Meaning	Depressive Symptoms
Interpersonal trust	−0.756 *** (0.163)	0.323 *** (0.081)	−0.592 *** (0.163)	0.318 *** (0.081)	−0.625 *** (0.158)
Intergenerational interaction frequency	−0.238 *** (0.043)	0.093 *** (0.022)	−0.191 *** (0.040)	0.090 *** (0.022)	−0.195 *** (0.041)
Life meaning			−0.505 *** (0.049)		−0.498 *** (0.048)
Digital social participation					−0.899 *** (0.201)
Interpersonal trust × Digital social participation					−0.719 ** (0.280)
Digital social interaction				0.112 *** (0.020)	
Intergenerational interaction frequency × Digital social interaction				−0.035 *** (0.009)	
Age	−0.004 (0.015)	−<0.001 (0.006)	−0.004 (0.015)	0.002 (0.006)	−0.028 * (0.014)
Gender (male)	−0.964 *** (0.164)	0.040 (0.074)	−0.944 *** (0.158)	0.022 (0.074)	−0.920 *** (0.157)
Hukou (urban)	−0.913 *** (0.211)	−0.012 (0.073)	−0.919 *** (0.196)	−0.050 (0.073)	−0.741 *** (0.201)
Marital status (ref. married)					
Divorced	0.469 (0.678)	0.049 (0.288)	0.494 (0.692)	0.018 (0.286)	0.561 (0.683)
Widowed	1.345 *** (0.260)	−0.207 * (0.119)	1.241 *** (0.241)	−0.204 * (0.117)	1.205 *** (0.238)
Never married	0.211 (1.085)	−0.432 (0.400)	−0.007 (1.032)	−0.358 (0.400)	−0.015 (1.047)
Cohabiting	1.734 ** (0.763)	−0.539 (0.612)	1.462 ** (0.685)	−0.554 (0.611)	1.515 ** (0.701)
Illness/injury severity	1.118 *** (0.079)	−0.157 *** (0.038)	1.038 *** (0.076)	−0.150 *** (0.039)	1.014 *** (0.078)
Life satisfaction	−1.005 *** (0.093)	1.070 *** (0.047)	−0.465 *** (0.104)	1.073 *** (0.046)	−0.487 *** (0.100)
_cons	19.324 *** (1.045)	2.788 *** (0.449)	20.731 *** (1.028)	2.576 *** (0.443)	22.750 *** (0.997)
*N*	4394	4394	4394	4394	4394
Prob > F	<0.001	<0.001	<0.001	<0.001	<0.001
R^2^	0.212	0.221	0.258	0.227	0.268

Note. Survey-weighted estimates with design-based standard errors in parentheses; *N* = 4394. *** *p* < 0.01, ** *p* < 0.05, * *p* < 0.1. Marital status is entered as dummy variables with married respondents as the reference category.

**Table 5:** Survey-weighted mediation analysis (*N* = 4394).

**Panel A.** Unconditional indirect associations					
**Variable**	**Effect**	**Coefficient**		**SE**	**95% CI**
Interpersonal trust	Total	−0.756		0.163	[−1.075, −0.436]
	Direct	−0.592		0.163	[−0.911, −0.274]
	Indirect	−0.163		0.046	[−0.252, −0.074]
	Proportion mediated	0.216		0.071	[0.078, 0.354]
Intergenerational interaction frequency	Total	−0.238		0.043	[−0.323, −0.153]
	Direct	−0.191		0.040	[−0.270, −0.112]
	Indirect	−0.047		0.013	[−0.072, −0.021]
	Proportion mediated	0.196		0.053	[0.093, 0.300]
**Panel B.** Conditional indirect associations via life meaning at values of digital social interaction (H4b)					
**Value of Digital Social Interaction**			**Indirect**		**95% CI**
0 (never)			−0.057		[−0.088, −0.030]
1 (once every few months)			−0.039		[−0.065, −0.017]
3 (2–3 times a month)			−0.003		[−0.031, 0.021]
Mean − 1 SD			−0.072		[−0.109, −0.038]
Mean + 1 SD			−0.019		[−0.044, 0.001]
Index of moderated mediation			0.018		[0.008, 0.029]
**Panel C.** Conditional association between interpersonal trust and depressive symptoms at values of digital social participation (H4a)					
**Value of Digital Social Participation**			**Association**		**95% CI**
0 (non-participation)			−0.289		[−0.732, 0.154]
1 (participation)			−1.008		[−1.390, −0.625]

Note. Survey-weighted estimates; *N* = 4394. Confidence intervals are bias-corrected from 5000 bootstrap replications resampling primary sampling units and agree with design-based (delta-method) intervals to two decimal places. Panel B reports the indirect association through life meaning at the observed values of digital social interaction; the interaction terms are constructed from mean-centered variables, so the values shown are rescaled to the original 0–6 scale. Panel C reports H4a, which moderates the direct association and does not constitute moderated mediation.

**Table 6:** Survey-weighted logistic regression results (odds ratios; *N* = 4394).

	Model 1	Model 2	Model 3	Model 4	Model 5
Variable	Depressed	Life Meaningful	Depressed	Life Meaningful	Depressed
Interpersonal trust	0.684 *** (0.065)	1.505 *** (0.130)	0.720 *** (0.072)	1.501 *** (0.133)	0.697 *** (0.070)
Intergenerational interaction frequency	0.897 *** (0.025)	1.167 *** (0.039)	0.917 *** (0.026)	1.156 *** (0.037)	0.915 *** (0.027)
Life meaningful			0.384 *** (0.047)		0.390 *** (0.049)
Digital social participation					0.729 ** (0.107)
Interpersonal trust × Digital social participation					0.676 ** (0.120)
Digital social interaction				1.220 *** (0.047)	
Intergenerational interaction frequency × Digital social interaction				0.962 ** (0.018)	
Cutoff	CES-D-8 ≥ 16	Life meaning ≥ 6	CES-D-8 ≥ 16	Life meaning ≥ 6	CES-D-8 ≥ 16
*n* above cutoff (weighted %)	1445 (29.55)	3577 (79.13)	1445 (29.55)	3577 (79.13)	1445 (29.55)
*N*	4394	4394	4394	4394	4394
Prob > F	<0.001	<0.001	<0.001	<0.001	<0.001

Note. Odds ratios from survey-weighted logistic regressions with design-based standard errors in parentheses; *N* = 4394. *** *p* < 0.01, ** *p* < 0.05. CES-D-8 scores (range 8–32) were dichotomised at ≥ 16 and life meaning (range 0–10) at ≥ 6. At these cutoffs 1445 respondents (31.62% unweighted; 29.55% weighted) were above the depression cutoff and 3577 (78.27% unweighted; 79.13% weighted) at or above the life-meaning cutoff. Life meaning ≥ 5 as an alternative cutoff classified 4266 respondents (93.35% unweighted; 93.97% weighted), and the pattern of results was unchanged. Model fit is reported as the design-based F test; McFadden’s pseudo-R^2^ is not a design-based statistic and is therefore not reported for the survey-weighted models.

## Data Availability

The data analyzed in this study are from the China Family Panel Studies (CFPS), publicly available through the Institute of Social Science Survey at Peking University. Data access requires registration and application through the CFPS official portal. The statistical code and derived variables used in this study are available from the corresponding author upon reasonable request.

## Abbreviations

The following abbreviations are used in this manuscript:

CES-D	Center for Epidemiologic Studies Depression Scale
CFPS	China Family Panel Studies
OLS	Ordinary Least Squares
VIF	Variance Inflation Factor
